# Current Landscape of Methods to Evaluate Antimicrobial Activity of Natural Extracts

**DOI:** 10.3390/molecules28031068

**Published:** 2023-01-20

**Authors:** Rebeca Gonzalez-Pastor, Saskya E. Carrera-Pacheco, Johana Zúñiga-Miranda, Cristina Rodríguez-Pólit, Arianna Mayorga-Ramos, Linda P. Guamán, Carlos Barba-Ostria

**Affiliations:** 1 Biomedical Research Center (CENBIO), Eugenio Espejo School of Health Sciences, Universidad UTE, Quito 170527, Ecuador; 2 School of Medicine, College of Health Sciences, Universidad San Francisco de Quito (USFQ), Quito 170901, Ecuador

**Keywords:** antimicrobial activity, antimicrobial resistance, pathogens, biological evaluation, natural products, bioactive compound, secondary metabolites

## Abstract

Natural extracts have been and continue to be used to treat a wide range of medical conditions, from infectious diseases to cancer, based on their convenience and therapeutic potential. Natural products derived from microbes, plants, and animals offer a broad variety of molecules and chemical compounds. Natural products are not only one of the most important sources for innovative drug development for animal and human health, but they are also an inspiration for synthetic biology and chemistry scientists towards the discovery of new bioactive compounds and pharmaceuticals. This is particularly relevant in the current context, where antimicrobial resistance has risen as a global health problem. Thus, efforts are being directed toward studying natural compounds’ chemical composition and bioactive potential to generate drugs with better efficacy and lower toxicity than existing molecules. Currently, a wide range of methodologies are used to analyze the *in vitro* activity of natural extracts to determine their suitability as antimicrobial agents. Despite traditional technologies being the most employed, technological advances have contributed to the implementation of methods able to circumvent issues related to analysis capacity, time, sensitivity, and reproducibility. This review produces an updated analysis of the conventional and current methods to evaluate the antimicrobial activity of natural compounds.

## 1. Introduction

Antimicrobial resistance (AMR) is a growing concern worldwide [1]. According to a recent study, an estimated 4.95 million people died from diseases associated with AMR in 2019 [2,3]. Furthermore, the accelerated global spread of multi-resistant bacteria is particularly distressing [4]. Hospital strains and those associated with foodborne diseases pose a risk due to the extensive use and misuse of antibiotics for human health and livestock [5,6,7]. Moreover, new antimicrobials that block drug-resistant pathogens are not being developed quickly enough [8,9,10].

In this context, natural products represent an immense source of biologically active components [11,12]. Both primary and secondary metabolites synthesized by mammalian and plant cells, as well as microorganisms, have influenced the development of effective treatments for an array of diseases and health conditions, including infectious diseases, inflammatory processes, and cancer [13,14,15]. Although technological development has enabled improved extraction and characterization techniques of natural compounds [16,17], screening strategies are not always capable of unwrapping the mechanism of the isolated compounds responsible for the combinatory effect [18]. Sometimes the active compound operates at a lower degree compared with the whole extract [19]. It is important to consider the sample solubility; for the extraction of hydrophilic and lipophilic compounds, solvents with a variety of polarity indexes, such as acetone, acetonitrile, dimethyl sulfoxide, ethanol, hexane, methanol, and dichloromethane, are commonly used [20,21,22]. Additionally, the effect of the selected solvent plays an important role in the extraction of total solids, phytochemical composition, and antioxidant potential, affecting the overall extraction efficiency of bioactive compounds [23].

In essence, the complexity of natural product mixtures and the lack of standardization for the procedures somewhat hinder the search for new antimicrobial drugs [24,25]. To generate cost-effective drugs based on natural compounds with better bioactive potential and fewer side effects, it is necessary to set up the proper assays to confirm their activity and establish their ranges and mechanisms of action [26,27,28,29]. In addition, further focus is required on the synergistic action of natural extract mixtures alone and employed in combination with available antimicrobials [30,31].

In terms of methods employed to evaluate antimicrobial activity, traditional technologies are the most widely used [32]. New evaluation techniques have been developed to overcome some of the disadvantages of classic methods, such as low reproducibility and lengthy processing times [11,33]; still, high costs and limited accessibility restrict these systems from frequent analysis, especially in resource-limited regions [34]. This review describes the most common *in vitro* assays currently used to characterize the antibiotic, antifungal, antiparasitic, and antiviral activities of promising natural compounds. In addition, each section presents the advantages and disadvantages of the discussed methodologies and includes emergent methods and future trends relevant to the field.

## 2. Antibacterial Activity

Several antibacterial susceptibility testing methods (AST) are available to determine bacterial susceptibility to antimicrobials. The selection of a method is based on many factors, such as practicality, flexibility, automation, cost, reproducibility, accuracy, and whether the results will be used for clinical or research purposes. AST methods must provide reproducible results in day-to-day laboratory analysis to be comparable with an acknowledged “gold standard” reference method [35]. Many authors have focused on plant and microbial metabolites as potential antibacterial agents. However, it is hard to compare these results because of the non-standardized techniques used for inoculum preparation and size, growth medium, incubation conditions, and endpoint determination [32]. The test organisms recommended by the Committee for Clinical Laboratory Standards (CLSI) in the preliminary screening for antibacterial activity are the Gram-positive *Enterococcus faecalis* (ATCC 29212) and *Staphylococcus aureus* (ATCC 29213) and the Gram-negative *Escherichia coli* (ATCC 27853) and *Pseudomonas aeruginosa* (ATCC 25922) [36].

The European Committee on Antimicrobial Susceptibility Testing (EUCAST) [37] and the CLSI guidelines are available to standardize *in vitro* AST methods related to clinical testing; these guidelines are also used for natural compounds since there are no standards of their own. However, natural extracts comprise a mixture of molecules that may not perform as expected in the test system. There are different challenges when using clinical guidelines for natural extracts. First, most antibiotics are hydrophilic, so AST methods are optimized for this condition, whereas natural extracts are lipophilic, meaning they are not fully soluble in water [38,39]. Another problem is the absence of the minimum drug concentration of natural compounds expected to be effective against bacteria (the breakpoint). Most of the studies rely on the minimal inhibitory concentration (MIC). MIC values range between 0.01–10 µg/mL for antibiotics, whereas plant extracts are considered antimicrobials if their MICs are between 100–1000 µg/mL [40]. Some authors even consider different cutoffs depending on the compound. For example, a concentration of 1000 µg/mL is considered the breakpoint for a polyphenol. This lack of standardization makes it difficult to have comparable and trusty results [38].

**Figure 1:**
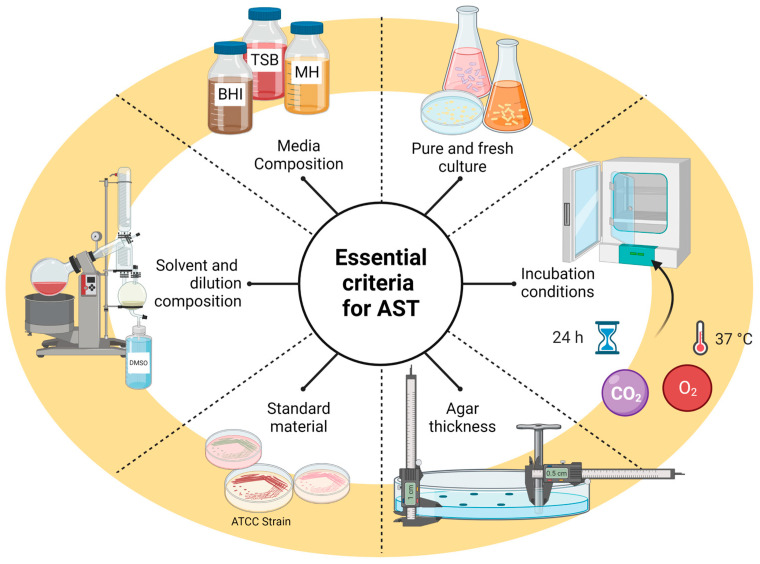
Key considerations to assess Antibacterial Susceptibility Tests (AST) in natural compounds; BHI: Bain heart infusion, TBS: Tryptic soy broth, MH: Mueller Hinton, ATCC: American Type Culture Collection, DMSO: Dimethyl sulfoxide.

With all these variables, no single method fits all-natural compounds evaluation but rather a combination of methods that consider particular characteristics and are best suited for assessing the sample. No matter which method is chosen, essential considerations must be taken into account to have reliable results, as shown in Figure 1.

This section will discuss the main characteristics of the standard methodologies used to assess the antibacterial activity of natural compounds.

### 2.1. Disk Well Diffusion

This is the most common qualitative assay for bacteria and fungi; due to its low cost and the fact that it does not require specialized laboratory facilities, it is used to screen many samples. In this technique, a paper disk or a well is filled with the extract, which spreads through the agar plate containing the bacteria of interest; then, the plate is incubated for 24–48 h [12,41]. The concentration is inversely related to the distance from the area or disk/well. The zone without bacterial growth around the disk/well is measured to compare with the antimicrobial used as a control [42,43]. The limitations include the differential diffusion of the extracts, such as essential oils containing terpenoids, which have limited solubility; therefore, they may not reflect the proper antibacterial activity, producing a false-negative result [44,45].

### 2.2. Agar Dilution Method

Agar dilution is a well-established method for determining antimicrobial susceptibility. The antimicrobial agent is incorporated into a series of agar plates containing the antimicrobial agent to be tested in increasing concentrations [46]. Inoculums of different microorganisms can be rapidly and simultaneously applied to the agar surface and incubated for 24–48 h. The standardized inoculum can be prepared by allowing the growth of the microorganism up to 0.5 turbidity on the McFarland scale (∼1 × 10^8^ colony forming units (CFU) mL^−^^1^). Growth is measured and compared with the control. One of the limitations is that when this method is used to assess the antibacterial activity of essential oils, it is difficult to make stable emulsions with the agar. Additionally, there is a problem with the concentration of the extract used since the agar may be too diluted to be solidified properly [47]. Mueller Hinton is generally used since it has proved to be the best of all available media for routine susceptibility testing of non-fastidious bacteria. For *Helicobacter* and *Haemophilus* species, supplementation with 5% fresh sheep blood is needed [48].

### 2.3. Broth Dilution Method

This is a more precise method to overcome the challenges regarding hydrophobic compound dilution in agar. Here, the tested compound is added to the culture medium where bacteria are grown; a sample is taken out every 10–20 min and diluted before being plated onto the agar, where colonies are counted after 24–48 h [49]. A variation of this method consists of using microtiter plates. Bacterial growth is measured spectrophotometrically through optical density (600 nm) or using cell viability indicators such as resazurin and methylthiazoldiphenyltetrazolium (MTT) [50,51]. However, when using colored natural extracts such as anthocyanins and carotenoids, they interfere with a correct turbidity measure. Another limitation is that this assay is not appropriate for oxygen-sensitive bacteria since incubation requires 150–200 rpm shaking most of the time [52]. A shared limitation with other methods remains in incorporating hydrophobic compounds into the media [42].

### 2.4. Thin-Layer Chromatography-Bioautography

Considering that natural extracts are mixtures of molecules, it is essential to have bioassay-guided fractionation methods where separation and biological activity are assessed simultaneously [53,54]. Thin-layer chromatography (TLC) is performed using whole extracts. The plate is sprayed or submerged into the bacterial suspension (direct bioautography) or covered with a layer of agar previously inoculated with the bacteria of interest (overlay bioautography). A positive result is considered when inhibition zones are observed after proper incubation times [53,54,55].

In summary, although various methods exist, the goal of *in vitro* antimicrobial susceptibility testing is the same: to provide a reliable predictor of how a microorganism is likely to respond to an antimicrobial extract. Therefore, emergent AST methods must include a great capacity to process the vast amount of natural extracts in a simple, cost-effective, reproducible way [56].

Additionally, fluorescent dyes are employed in a variety of methods as a quick way to identify bacterial populations and assess cell viability [57]. This assay can produce a rapid, thorough, and measurable overview of the bacterial population. Although these procedures can be validated to meet the International Organization for Standardization (ISO) standards, flow cytometry needs a significant initial investment and substantial training to calibrate the equipment, set up complex assays, and analyze data and results [58]. Specifically for natural products, a recent publication was used to determine antibacterial activity in citric oil samples using a series of systematic standardization steps to find appropriate conditions for flow cytometry (acquisition of bacteria, fluorochrome concentration, determination of suitable comparison antibiotics, and exposure times) [59].

### 2.5. Molecular Methods

Other approaches to assess antibacterial activity that are becoming more widely employed do not require culturing bacteria. Due to its lower cost and capacity to produce quick results, quantitative polymerase chain reaction (qPCR) is currently utilized to evaluate the efficacy of antimicrobial substances and methods. qPCR can identify and quantify the amount of target genetic material present in a sample without culturing cells. To differentiate living cells from dead ones, a viability polymerase chain reaction (PCR) assay can be used. This entails incubating samples with a DNA-binding dye, such as propidium monoazide (PMA), which binds to free DNA (including dead cells with broken membranes) and inhibits the PCR amplification, so living cells are the ones detected and amplified [58]. Following the development of molecular techniques such as PCR, sequencing, and metagenomics, many studies have favored these cutting-edge methods at the expense of bacterial culture. Nevertheless, metagenomics presents some drawbacks, notably, a depth bias due to the low sensitivity of some of the primers used [60]. Most current methods only detect DNA and, in some cases, cannot distinguish between DNA from living and dead bacteria and the DNA from the transient bacteria under study. As a result, advanced culture techniques are being developed. In recent years, new culture mediums and conditions have enabled the development of culturomics, a high-throughput culture approach [61]. According to some experts, this demonstrates that culture media continue to be an essential technique for bacteriologists to isolate commensal and harmful bacteria. Therefore, there are still many advancements in bacterial culture that will expand the bacterial repertoire and better comprehend certain diseases [62]. For MIC determination using molecular methods, DNA samples are taken after exposing the cells to treatments with higher concentrations of the natural extracts and using commercial antibiotics as controls [63].

## 3. Antifungal Activity

Infections caused by fungal pathogens are a global threat and affect more than a billion people, resulting in over 300 million people with invasive fungal infection (IFI) and more than 1.7 million deaths annually, which is equivalent to that caused by tuberculosis and more than triple that of malaria [64,65]. IFIs need appropriate antifungal therapy, but only a few antifungal drugs are available [66]. In addition, many have significant drawbacks, such as high cell toxicity, undesirable interactions, poor pharmacokinetics, and a narrow spectrum of activity [67]. Furthermore, in recent decades, the number of patients with risk factors for IFI (severe immunosuppression, exposure to long courses of broad-spectrum antibiotics, and implanted medical devices) has increased [68]. The rising incidence of IFIs [68] poses even more pressure on health systems since acquired resistance has emerged in fungal pathogens, hampering the treatment of severe patients [66,69,70]. In this complex scenario, including changes in patient factors and the lack of effective treatment options, it is critical to identify novel compounds with antifungal activity.

**Figure 2:**
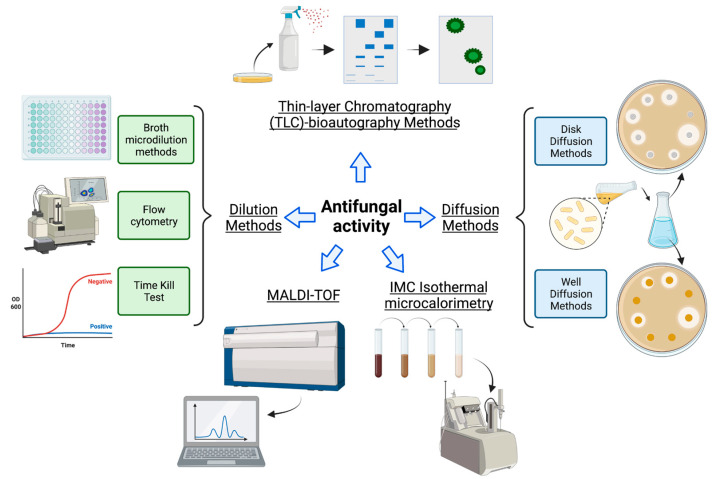
Summary of the principal methods used to evaluate antifungal activity; MALDI-TOF: matrix-assisted laser desorption/ionization-time of flight; OD: optical density.

Due to its broad applicability among microorganisms, some basic principles of antibacterial assays, such as dilution and diffusion, are also used to evaluate antifungal activity. Nevertheless, due to the particular features of the fungal lifestyle, some of the modifications listed in Table 1 should be made to properly evaluate the *in vitro* antifungal activity. Similar to antibacterial tests, the diameter of growth inhibition in solid media is the key parameter for evaluating the antifungal activity of natural compounds or extracts. In the case of microdilution, cells are plated after incubation with different concentrations of the extract/compound (Figure 2). The effect is determined by comparing the growth of treated cells with that of the corresponding untreated control. Although these methods help evaluate the activity of pure compounds or natural extracts, the standard procedure has to be modified to successfully evaluate the activity of natural extracts due to differences between both types of samples (water solubility, purity, and concentration of the bioactive molecule). It is important to consider these factors to correctly interpret the results.

The time-kill method is another simple and effective assay for evaluating the antifungal activity by measuring the effect of pure compounds or natural extracts on fungal growth, measured by changes in cell density (Figure 2). It should be mentioned that time-kill is not designed to exclusively measure antifungal activity; the same approach is commonly used for measuring antibacterial and other antimicrobial activity. This method provides additional details on the type of effect (fungistatic or fungicide) of the analyzed compounds. Additionally, together with the microdilution test, it allows for calculating the minimum inhibitory concentration (MIC). An antifungal activity assay using flow cytometry (FC) or fluorescence-activated cell sorting (FACS) is based on light scattering and fluorescence emission by fluorescently labeled fungal cells passing through a laser beam [68]. Alterations in the fluorescence distribution of the population are interpreted as changes in cell viability and, thus, as an indication of antifungal activity (Figure 2). Although it is not the most common method, FC and FACS can also be used to determine the MIC [68].

Matrix-assisted laser desorption/ionization-time of flight (MALDI-TOF) is a mass spectrometry ionization technique that uses laser energy to create ions from large molecules, such as proteins. This technique has been mainly used to determine the antifungal activity of pure molecules, but due to its versatility [71,72], it holds potential for evaluating the antifungal activity of natural extracts. Isothermal microcalorimetry (IMC) allows real-time, continuous measurement of the heat flow and the evaluation of heat changes in a sample. However, these two methods are used less frequently due to the need for specialized equipment and the fact that they are more time-consuming or strenuous, even though results are obtained quickly [73]. Table 1 summarizes the key points of the methods and modifications used to evaluate the antifungal activity and other less common but equally effective alternatives.

Mainly because of the threat caused by the worldwide rise of fungal infections, it is highly relevant to develop simpler, faster, more robust, and high-throughput methods that allow the evaluation of multiple extracts/compounds against multiple fungal pathogens in a simple manner.

**Table 1:** Methods to assess antifungal activity.

Method/Type of Assay		Description	Media and Microorganism	Advantages	Disadvantages	Ref.
Diffusion methods	Disk/well diffusion	- Disks or wells containing the extract - Diameter of the inhibition halo is measured - The larger the diameter, the better the activity	- Sabouraud-dextrose agar. Tryptone-yeast-glucose agar - Agar potato-dextrose agar - Yeasts and filamentous fungi	- Simplicity - Low cost - Up to 6 extracts per plate can be screened	- Not appropriate for testing non-polar samples - Challenging to run on high-throughput screening systems	[74,75]
Dilution methods	Broth microdilution	- Incubation with different concentrations of the extract - Plating and counting colonies in agar - A lower number of colonies indicates a stronger activity	- Sabouraud dextrose broth - RPMI 1640 growth medium with glucose - Filamentous fungi and yeasts	- Gold standard - MIC can be calculated	- Insoluble samples may interfere with readings - Labor-intensive - Modification is needed when the fungus is growing as hyphae	[76,77,78]
	Time-kill test	- Sampling of control (cells, no drug) and antimicrobial agent-containing cultures at intervals (usually 0, 4, 8, 10 to 12, and 24 h of incubation) - Survivor colony count (CFU per milliliter) determined by spreading onto agar plates - Lower number of colonies indicates a stronger activity	- RPMI 1640 growth medium with glucose - Yeast extract Peptone Dextrose - Filamentous fungi and yeasts	- MIC can be calculated - Synergistic or antagonistic interactions can be evaluated	- Modification is needed when the fungus is growing as hyphae - Labor-intensive	[73,79,80]
	Flow cytometry, or fluorescence-activated cell sorting (FACS)	- Incubation with different extract concentrations - Dilution, staining, and fluorescence measurement by flow cytometer - A lower fluorescence indicates better activity	- RPMI - Filamentous fungi and yeasts	- MIC can be calculated - Highly sensitive - Quick results	- Labor-intensive - Requires a high level of technician expertise - Specialized equipment required - Time-consuming.	[68,73,81,82]
Calorimetry	Isothermal microcalorimetry (IMC)	- Serial dilutions of the plant extract with fungal cells - Growth is monitored by changes in the total heat - Lower total heat indicates better activity	- RPMI - Yeasts - *Candida* - *Rhizopus* - *Fusarium*	- Compatible with high throughput - Routine clinical testing and antifungal drug discovery	- Specialized equipment required - Specialized and trained personnel required	[83]
Mass Spectrometry	MALDI-TOF	- Incubation of fungal cells with the plant extract - Growth is monitored by proteome changes (MALDI-TOF) compared with an untreated control	- RPMI - Filamentous fungi and yeasts	- Elimination of subjectivity present in the visual readout - Highly sensitive	- Expensive - Requires specialized equipment and training	[84,85]
Thin-layer chromatography (TLC)	Agar overlay bioautography	- Inoculation of fungal cells in melted agar - Mixture applied to TLC plate embedded with the plant extract - Incubation and staining of inhibition bands	- Sabouraud - Malt agar - Filamentous fungi and yeasts	- Consistent with spore-producing fungi - Inexpensive - Simple	- Qualitative - Difficulties in obtaining complete contact between the agar and the plate	[53,86,87]

MALDI-TOF: Matrix-Assisted Laser Desorption/Ionization Time-of-Flight; MS: mass spectrometry; RPMI: Roswell Park Memorial Institute Medium. MIC: Minimum inhibitory concentration; CFU: colony-forming unit.

## 4. Antiparasitic Activity

Parasitic infections are caused by microorganisms that thrive at the expense of their host, causing significant morbidity and mortality worldwide [88]. Most human parasitic diseases predominate in developing countries and have been considered part of the group of neglected tropical diseases [89]. Given their broad definition, these organisms can range from single–cell eukaryotes to complex multicellular organisms such as endoparasites and ectoparasites [90]. Therefore, the identification of new drugs to treat parasitic diseases is a major priority. Unfortunately, the discovery rate for novel antiparasitic compounds has decreased dramatically in the past couple of decades [89]. Nonetheless, plant extracts have always been described in the literature as a new source of secondary metabolites and potential antiparasitics [91,92]. This section will focus on the methods commonly used to detect antiparasitic activity on endoparasites such as protozoa or helminths, which cause a significant burden in developing countries.

### 4.1. Antiprotozoal Activity

**Figure 3:**
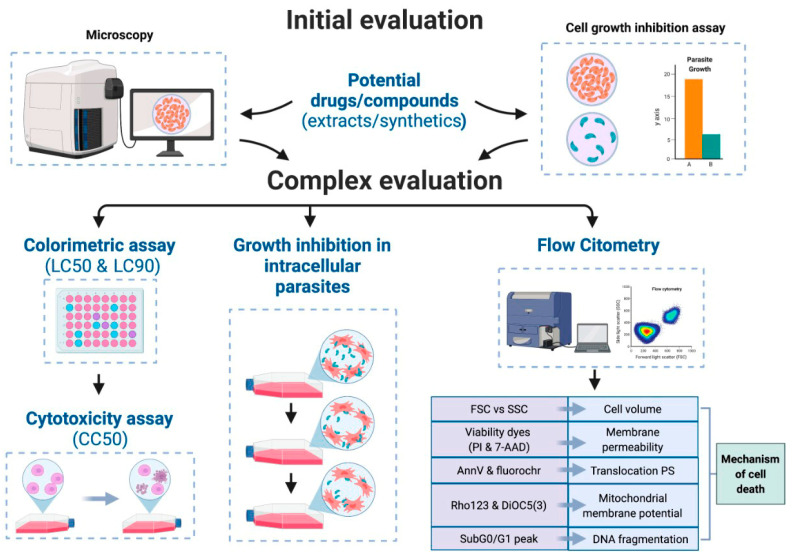
Process of *in vitro* evaluation for potential antiprotozoal activity; LC: Lethal concentration, CC50: 50% cytotoxicity concentration, FSC: Forward Scattered FSC, SSC: Side Scatter, PI: Propidium iodide, 7-AAD: 7-aminoactinomycin D, AnnV: Annexin V, PS: phosphatidylserine, Rho123: Rhodamine 123, DiOC5(3): 3,3′- dipentyloxacarbocyanine iodide, SubG0 and G1: cell cycle phases. In Cell growth inhibition assay, A and B represent the untreated and treated samples of the assay.

In the search for novel antimicrobials and antiparasitic drugs, the first screening of all candidate compounds and extracts is usually assessed by a range of classic and standardized assays [93]. More complex evaluations can further assess the test compounds with the best potential (Figure 3). In clinical samples, microscopic examination is the gold standard for unicellular parasite identification [94]. As a result of its simplicity, microscopy can offer preliminary data regarding morphological alterations in parasitic cells after applying the potential antiparasitic compound in the culture. Death by apoptosis can be characterized by a shrinkage of the cell or loss of cellular volume [95]. In necrotic cell death, the parasites could undergo cytolysis, which can be visualized as small fragments or cell debris [96].

Colorimetric assays can also support antiparasitic studies by assessing cell viability and determining the LC50 (lethal dose, 50%) and LC90 (lethal dose, 90%) [97]. These assays are similar to the colorimetric techniques used in mammalian cell cytotoxicity assays, as shown in Table 2. Flow cytometry is another valuable tool that allows the simultaneous multi-parameter analysis of single cells [98]. Subsequently, the confluence of this data can provide valuable information to characterize the mechanism of cell death prompted by the candidate compound. Furthermore, cell proliferation assays allow the adjustment and evaluation of different parameters, such as extract concentration and measurement over multiple time points. Several protozoans undergo their life cycle inside the host cells; therefore, it is also essential to assess the test compound via intracellular parasite proliferation assays [99].

In recent years, novel techniques have gained more notoriety and provided insightful contributions to antiparasitic drug discovery. Current and future trends for antiparasitic drug discovery take advantage of in silico models as a valuable tool for initial screenings before further lab analysis [121,122,123]. Subsequently, the electron microscope has a growing role in characterizing proteins, subcellular targets, and drug action mechanisms in parasitic cells during structure-based drug design [124,125].

In leishmaniasis drug discovery, drugs that target specific parasite–host interactions or vector–host interactions have seen a growing interest [123]. Consequently, their evaluation demands other essays related to the analysis of the immunomodulatory properties of the candidate compounds or their transmission-blocking strategies [126]. Another current method is the adenosine triphosphate (ATP) bioluminescence assay, which is usually used to measure the ability to produce ATP by bacteria or fungi (including yeast and molds) [127,128]. In addition, it is also used to evaluate the influence of biofilms in situ and for medication screening on Leishmania [32].

For Chagas disease, recent advances have taken advantage of next-generation sequencing or high-throughput sequencing for phenotypic-based drug discovery [129]. Moreover, other technological developments show great potential to accelerate initial screenings of multiple compounds [124] quickly. For example, automated microscopy has also significantly influenced drug discovery. It can increase screening throughput by replacing laborious and subjective manual microscopic observation and creating algorithmic systems to identify morphological injury and changes in target parasitic cells [130]. Additionally, omics-based techniques allow the determination of novel targets for parasitic protozoan infections, including *Plasmodium spp.*, *Toxoplasma spp*., *Trypanosoma spp*., and *Leishmania spp*. [131].

### 4.2. Anthelmintic Activity

Active extracts or pharmaceuticals used against helminths are named anthelmintic; these drugs are used to regulate the uncontrollable reproduction of worms and are expected to eliminate the parasites either by killing them or stimulating their escape from the infected organism [132]. Unlike antiprotozoal activity, microscopy’s observation is the gold standard for identifying anthelmintic activity. This examination relies on the absence of movement (paralysis or death) of larvae or adult worms, the inability of the eggs to hatch, or the larvae to continue with their life cycle [133]. These observations indicate the effectiveness of a given extract or compound in inhibiting parasitic growth through various mechanisms, including alterations in metabolism [134].

Current trends for assessing the anthelmintic activity of natural extracts are based on *in vitro* physiological assays. At different stages of its life cycle, the parasite is cultured in the presence of the tested compound or extract concentrations, and its viability, motility, and growth are evaluated [135]. A list of the most common tests carried out for anthelmintic activity is described in Table 3.

**Figure 4:**
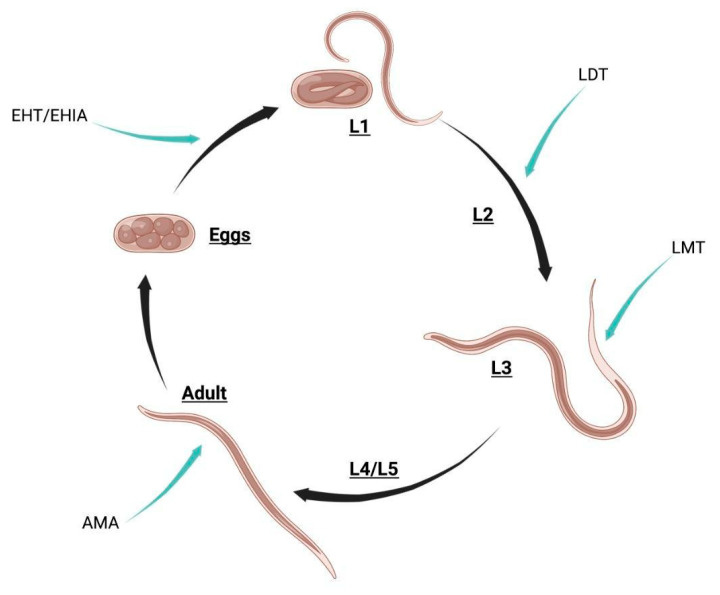
Anthelmintic evaluation throughout the parasite life cycle. L1 to L5 refer to larval stages. EHT/EHIA: Eggs Hatching Test/Eggs Hatching Inhibition Assay, LTD: Larval Development Test, LMT: Larval Motility Test, AMA: Adult Motility Assay.

As mentioned before, these physiology-based assays can be performed at different stages of the parasite life cycle. Figure 4 illustrates the type of tests performed along the parasite life cycle.

Results from current studies suggest that plant extracts have compounds with anthelmintic properties that can be further studied for antimicrobial activity and drug discovery [135,143]. Therefore, it is recommended that anthelmintic tests be combined with other antimicrobial screenings, such as the ones presented in this review.

A new approach to studying anthelmintic activity employs a high-throughput screening platform using the model organism *Caenorhabditis elegans*; here, new compounds with anthelmintic properties are identified by forward and reverse genetics approaches [144]. The advantage of using *C. elegans* to uncover new possible anthelmintics is that this organism has a broad genetic toolset, which facilitates the investigation of the mode of action for potential drug candidates [144,145]. Future trends focus on the development of automated imaging and computational frameworks for the analysis of subtle changes in worms’ phenotype induced by test compounds that may be valuable in determining their antiparasitic effects [146].

## 5. Antiviral Activity

**Figure 5:**
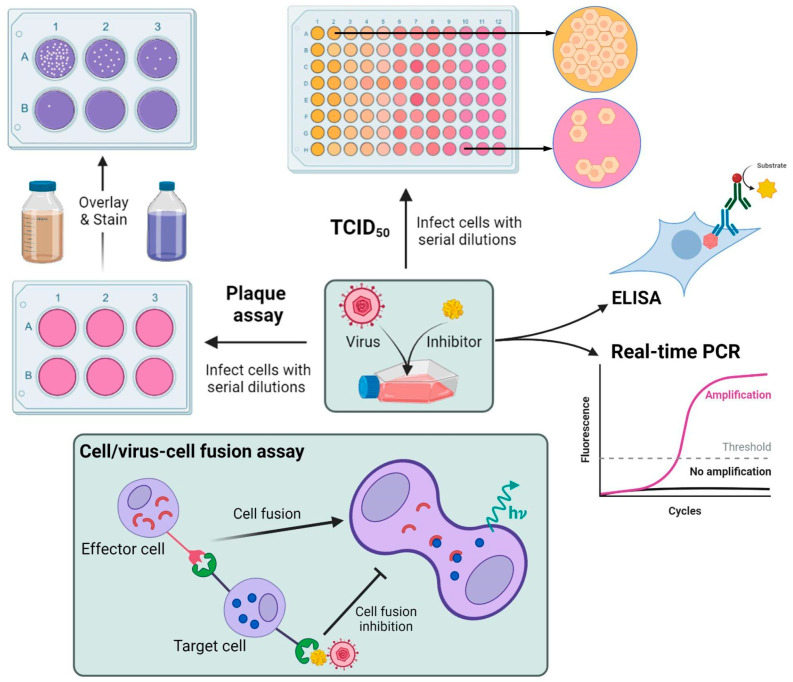
Common cell-based *in vitro* assays for direct and indirect antiviral activity determination of natural molecules. TCID50: 50% tissue culture infectious dose assay; ELISA: enzyme-linked immunosorbent assay; PCR: real-time polymerase chain reaction; hν: emitted energy (fluorescence, luminescence).

Antiviral screening has gained substantial attention since the SARS-CoV-2 pandemic started in 2020. Several emerging and re-emerging viral infections cannot be prevented by vaccination or treated with antiviral drugs or monoclonal antibodies, such as viral diseases caused by the Zika virus, Dengue virus, and Chikungunya virus, among others [147]. In addition, due to the flexibility and adaptability of viruses that result in the development of drug-resistant mutants, there is a constant need to evaluate new inhibitors that can be incorporated into the drug discovery pipeline [148]. This section describes the most common cell-based assays used to evaluate antiviral activity (Figure 5).

Antiviral compounds generally target several key steps of viral life cycles: entry, replication, transcription or retrotranscription, proteolytic processing, and viral particle release [149]. Thus, the goal is to identify compounds that specifically inhibit viral or cellular targets essential for viral replication [150]. In this context, the evaluation of natural products is particularly appealing based on their biological importance and chemical and structural diversity. Plant extracts, triterpenoids, alkaloids, phenols, flavonoids, and honey are among the most analyzed natural extracts with antiviral activity [151,152].

Among the well-accepted methods used to screen for inhibitors aiming at the entire viral life cycle, the yield reduction assay remains a powerful technique [153,154]. The assay involves the infection of target cells in the presence of different concentrations of the test molecule, the collection of supernatants and cells after a cycle of virus replication, and the determination of the virus titers.

The two most commonly used methods to quantify the concentration of replication-competent virions are the plaque assay, which remains the gold standard, and the 50% tissue culture infectious dose assay (TCID50) [155,156,157]. Both assays depend on the capability to evaluate visible cytopathic effects in cell monolayers after the infection of susceptible cell lines. In the plaque assay, a confluent monolayer of host cells is infected with serial dilutions of the virus, and after adsorption, a semi-solid or solid medium is added on top of the monolayer to restrict virus diffusion at the sites of initial infection [158,159]. The local replication of the virus results in zones of cell death, termed “plaques”. Generally, cells are stained to enhance the contrast of the plaques and facilitate counting, and the titer is measured by the number of plaque-forming units per mL (PFU/mL) [160]. In contrast, the TCID50 endpoint dilution assay measures visible cell death inside each well of a 96-well tissue culture plate. The titer is calculated as the dose at which a virus sample is expected to infect a tissue or cell culture 50% of the time and is transformed into virus concentration/mL [161,162].

However, estimation of the relative infectivity by visual inspection can be complicated by variations in plaque size and irregular morphology; it can also be incompatible for non-lytic viruses. For those cases, the focus-forming assay (FFA) allows the identification of single foci by detecting virally encoded proteins expressed by infected cells. The viral titer is expressed in focus-forming units per mL (FFU/mL) [163,164]. Additionally, with the advancement of instrumentation and the high contrast of reagents, counting can be automated for more precision and less subjectivity in a high-throughput setting [163,164,165]. Additionally, other quick and sensitive methods, such as cell-based enzyme-linked immunosorbent assay (ELISA) and quantitative real-time polymerase chain reaction (PCR), are used to determine antiviral activity by measuring the reduction in viral antigen or virus nucleic acid in infected cells in the presence of the test molecule [166,167,168]. Similarly, transmission electron microscopy (TEM) can be used to quantify infectious particles and assess the potential antiviral activity of test compounds [169]. Nonetheless, analysis carried out by these methods may include non-infectious particles that do not contain genetic information, or rather all genetic material present in a sample, including extra genomes not packaged into virions [170].

Instead of aiming at the entire viral life cycle, other cell-based assays have been developed to screen viral inhibitors that target specific steps of the infectious process [171,172,173]. For instance, classic methods to screen inhibitors of viral entry include cell–cell fusion assays and cell–virus fusion assays. These methods use effector cells that express the viral entry protein, or recombinant virions, to facilitate fusion with target cells that express the host-cell receptor and carry a reporter system. The shift in the expression of the reporter corresponds to the efficacy of fusion inhibition [174,175,176]. Apart from blocking virus attachment and entry, other inhibitors typically target the components that are critical for viral genome replication. Due to polymerases being the preferred target for antiviral intervention [177,178], fluorescence-based quantitative real-time PCR and quantitative real-time reverse transcription PCR are the methods of choice to specifically monitor the activity of DNA and RNA polymerases in the presence of inhibitors [179,180,181].

The SARS-CoV-2 pandemic highlighted insufficient preparation for handling certain virus outbreaks. Although there has been extensive development in molecular and cellular biology methods and imaging techniques applied to antiviral testing, the assays developed are often limited by the availability of reagents and equipment that can be used in biosafety containment facilities [147]. Therefore, future trends are focused on the development of accurate, simplified, and fast cell-based assays for sensitive and quick high-throughput screening (HTS) of antivirals [182,183,184,185,186,187]. An additional complication encountered when testing natural products as antiviral agents is the tedious nature of identifying bioactive components, leaving the mechanism of action of the crude extract or the metabolites unknown [151]. In this regard, advances in in silico approaches have revealed a plethora of natural compounds that interact with viral protein targets and can be subjected to *in vitro* assays [188].

## 6. Conclusions and Future Perspective

The rising prevalence of drug-resistant diseases urges the use of standardized modern analytical technologies to detect and isolate novel bioactive chemicals from natural sources. Compounds produced by animals, plants, and microbes could offer new and simpler ways to combat harmful microorganisms. Unfortunately, the current range of approved antibacterial compounds from plants does not adequately reflect their potential for future use as antimicrobial agents [42]. Moreover, many promising medicinal plants and active compounds are yet to be studied phytochemically and pharmacologically [189,190].

As described in this article, a wide range of laboratory methodologies are currently used to analyze the antimicrobial activity of natural extracts. Overall, the assessment of most biological activities would benefit from using a combination of multiple assays that can complement or confirm the identification of the antimicrobial mechanisms involved. Although the most popular are the classic, basic methodologies, advanced biotechnological, genomics, proteomics, and metabolomics approaches are now used in natural extract research to assess antimicrobial activity and promote the development of new safe antimicrobial drugs [42,189]. It deserves to be pointed out that natural extracts have a complex range of metabolites, some of which can interfere with the wavelength maxima of the product during spectroscopic measurements because they are often intensely colored. In addition, the opacity of the solutions could interfere with the turbidity of bacterial growth [39,191,192]. In this sense, progress in computational chemistry, the use of public databases, and the implementation of in silico models to accurately assess the efficacy of a promising compound accelerate initial screenings [193,194]. For instance, the antibiotics and Secondary Metabolite Analysis Shell (antiSMASH) tool is a platform that allows the mining of antibiotic biosynthetic gene clusters (BGC) based on similarities to existing instances from plants, fungi, and bacteria [195]. Another new portal mines the genes that code for a natural product’s production and uses that information to see if other strains can manufacture specific critical intermediates of the natural product mining with, a tight emphasis on genes involved in the final few steps of biosynthetic pathways [196]. Furthermore, the implementation of advanced microscopy and high-throughput sequencing enables the analysis of drug action mechanisms and added properties related to the interactions between organisms with lower variability.

In summary, as technology advances and the world becomes increasingly automated in every aspect of life, methods that are regularly utilized, as well as other remarkably rapid and automated testing systems, will become standardized, more available, and more user-friendly. Furthermore, manual methods and automated systems for investigating antimicrobial activity must continue to be improved and updated for this purpose. In the meantime, a combination of manual and semi-automated testing procedures must be used to produce reliable results.

## Figures and Tables

**Table 2:** Methods of evaluation for antiparasitic activity in unicellular parasites (protozoa).

Type of Assay	Description Detection/Equipment	Activity Under Evaluation	Advantages	Disadvantages	Ref.
**Dye Exclusion**					
Resazurin	Colorimetric/M Determine the viability of cells by measuring the chromogenic reaction concentration of colored compounds in a solution	Metabolic activity. Mitochondrial dehydrogenase and oxidoreductase activity	- Cell-to-cell count (quantitative) - Simple - Inexpensive - Fast	- Counting errors - Difficulty in processing large numbers of samples	[100,101,102]
Tetrazolium salts (MTT, XTT, MTS, WST)			- Easy to use - Safe - High reproducibility	- Possible high background (interference with reagent/media) - Time-consuming - Record additional data about progressive cytotoxic effects)	[103,104,105]
**Flow Cytometry**					
FSC vs SSC	FC Variations in Forward Scattered (FSC) light determine volume changes and variations in Side Scatter (SSC) can determine internal composition	Cell size and volume	- Fully automated - High throughput data acquisition (data virtually stored) - Allows re-analysis	- Use of specific equipment - Cell lysis can create debris which may interfere with the results	[106,107]
Viability dyes PI, 7-AAD	Fluorescent/FC Loss of the integrity of the membrane can be determined by using “viability dies”	Cell cycle arrest Apoptosis/Necrosis Evaluation of membrane integrity	- Single-cell quantification of stained DNA	- Endpoint (fixed-permeabilized cells) - High throughput data acquisition (data virtually stored)	[108,109,110]
Annexin V (AnnV) conjugated to fluorochromes (FITC, PE, APC, etc.)	Fluorescent/FC Apoptotic cells show the migration of PS from the inner layer of the plasma membrane towards the outer layer (become exposed)	Translocation of phosphatidylserine (PS) Evaluation of membrane integrity	- Fully automated - High sensitivity	- Difficult to differentiate apoptotic from necrotic cells	[111,112,113]
Permeable cationic lipophilic fluorochrome -(Rho 123) and DiOC5(3)	Fluorescent/FC Apoptotic cells show alterations in the synthesis of ATP and the mitochondrial electron transport chain in the mitochondrial membrane	Mitochondrial membrane Potential	- Fully automated - Extremely low background - Immediate readout - Detection in mixed cell culture model	- Endpoint, need for cell engineering, decrease in luminescent signal with increased cell death, - Challenging to observe small changes in the number of dead cells.	[114,115]
Sub-G0/G1	Fluorescent/M Percentage quantification of cells with fragmented DNA by analyzing the “sub-G0/G1” peak in a DNA histogram	DNA fragmentation (strand breaks)	- Rapid - Detect cumulative apoptosis - Applicable to all cell types	- Low detection rate: - If cells enter apoptosis from the S or G2/M phase of the cell cycle - If there is an aneuploid population undergoing apoptosis	[116,117]
***In Vitro* culture**					
Growth Inhibition Assay	Colorimetric/M The candidate drug is added to the culture, and the parasite grows during a determined time. Variables: drug concentration and time collection points	Cell viability/proliferation	- Live cell analysis - Long experiments - Several options to visualize the results by different detection methods.	- Requires specific equipment - Expensive reagents - May require damage to the cells for quantification (toxicity from the reagents or cell fixation) - Limited in intracellular parasites	[118]
Intracellular Parasite Growth Inhibition Assay	Colorimetric/M Eukaryotic cells are infected with intracellular parasites *in vitro*. Then, test compounds are added, and the culture grows during a determined time	Cell viability/proliferation inside host	- Cell-to-cell count and estimated number of viable cells (quantitative) - Simple - Inexpensive	- Requires the proper growth of the host eukaryotic cells each time - Affected reproducibility between results/replicates	[119,120]

M: microscopy, FC: flow cytometry, APC: Allophycocyanin, FITC: Fluorescein isothiocyanate PI: Propidium iodide, 7-AAD: 7-aminoactinomycin D, MTT: 3-(4,5-dimethylthiazol-2-yl)-2,5-diphenyltetrazolium bromide), XTT: (2,3-bis(2-methoxy-4-nitro-5-sulfophenyl)-5-carboxanilide-2*H*-tetrazolium), MTS: 3-(4,5-dimethylthiazol-2-yl)-5(3-carboxymethonyphenol)-2-(4-sulfophenyl)-2*H*-tetrazolium, PE: Phycoerythrin, WST: Water-soluble tetrazolium salts, Rho 123: Rhodamine 123 and DiOC5(3): 3,3′- dipentyloxacarbocyanine iodide, Sub-G0, G1, S, G2 and M: cell cycle phases.

**Table 3:** Most common methods for *in vitro* determination of anthelmintic activity.

Assay	Description	Activity Under Evaluation	Advantages	Disadvantages	Ref.
Adult Motility Assay (AMA)	- Adult worms are subjected to different concentrations of the extract - The time of paralysis (lack of movement) and the time of death are measured	Inhibition of adult worm motility, which can indicate mortality or paralysis	- Simple - Low cost - Earthworms can be used instead of gastrointestinal parasites due to their anatomical and physiological similarities	- Cannot tell the drug’s mode of action - Can take several days to track the recovery or death of the worms	[133,134,136]
Larval development test (LDT)	- L1 larvae are subjected to different concentrations of the extract and allow to grow several days - Larvae that reach L3 stage or survive are counted under the microscope and registered	Development of L1 to infective L3 larvae	- Simple - Low cost - Anthelmintic resistance can be measured with specific equipment	- Fresh L1 larvae have to be grown from eggs - Requires time to track the development of the larvae from L1 to L3	[137,138]
Larval mortality/paralysis test (LMT)	- L3 larvae are subjected to different concentrations of the extract and allow to grow 24 h - Motionless larvae L3 are counted under the microscope and registered	Inhibition of L3 larvae motility	- Simple - Low cost	Motile L3 larvae have to be grown prior testing	[139]
Egg hatch test (EHT) or Egg hatch inhibition assay (EHIA)	- Parasite eggs are isolated from freshly collected feces - Eggs are subjected to different concentrations of the extract and allowed to hatch for 48 h - The addition of helminthological iodine stops hatching - Eggs and L1 larvae are counted under the microscope	Inhibition of eggs hatching	- Low cost - Can be performed in microtiter plates	Eggs have to be isolated from infected samples or adult females	[133,138,140,141,142]

L1 to L3 refer to larval stages.

## Data Availability

All tables are created by the authors. All sources of information are adequately referenced. There is no need to obtain copyright permissions.
